# Uncovering the Gene Regulatory Network of Endothelial Cells in Mouse Duchenne Muscular Dystrophy: Insights from Single-Nuclei RNA Sequencing Analysis

**DOI:** 10.3390/biology12030422

**Published:** 2023-03-10

**Authors:** Yan Shen, Il-man Kim, Mark Hamrick, Yaoliang Tang

**Affiliations:** 1 Medical College of Georgia, Augusta University, Augusta, GA 30912, USA; 2 Anatomy, Cell Biology, and Physiology, School of Medicine, Indiana University, Indianapolis, IN 46202, USA

**Keywords:** cell metabolism, Duchenne muscular dystrophy, *dystrophin*, single-nuclear RNA sequencing (snRNA-seq), skeletal muscle-derived endothelial cells

## Abstract

**Simple Summary:**

Duchenne muscular dystrophy is a devastating disease that results from mutations in the dystrophin gene (DMD) and can lead to heart and respiratory failure. Despite the critical role of endothelial cells (ECs) in disease progression, there is limited understanding of the impact of the DMD gene on the gene regulatory network of ECs. In our study, we aimed to fill this knowledge gap by utilizing single-nuclei RNA sequencing (snRNA-seq) to evaluate the transcriptomic profile of ECs from skeletal muscle in DMD mutant mice and wild-type control mice. Our results showed that the DMD mutation resulted in the upregulation of multiple long noncoding RNAs (LncRNAs). Additionally, we found that the metabolic pathway activity of ECs was altered, with a decrease in oxidative phosphorylation, glycolysis, and pyruvate metabolism and an increase in purine metabolism and pyrimidine metabolism. Overall, our study provides new insights into the gene regulatory program in ECs in DMD and highlights the importance of further research in this area. The results of this study have important implications for the development of therapeutic strategies for patients with DMD.

**Abstract:**

Introduction: Duchenne muscular dystrophy (DMD) is a severe X-linked recessive disorder caused by mutations in the *dystrophin* gene, which leads to heart and respiratory failure. Despite the critical impact of DMD on endothelial cells (ECs), there is limited understanding of its effect on the endothelial gene network. The aim of this study was to investigate the impact of DMD on the gene regulatory network of ECs. Methods and Results: To gain insights into the role of the *dystrophin muscular dystrophy* gene (*DMD*) in ECs from Duchenne muscular dystrophy; the study utilized single-nuclei RNA sequencing (snRNA-seq) to evaluate the transcriptomic profile of ECs from skeletal muscles in *DMD* mutant mice (*DMD^mut^*) and wild-type control mice. The analysis showed that the *DMD* mutation resulted in the suppression of several genes, including SPTBN1 and the upregulation of multiple long noncoding RNAs (lncRNAs). GM48099, GM19951, and GM15564 were consistently upregulated in ECs and skeletal muscle cells from *DMD^mut^*, indicating that these dysregulated lncRNAs are conserved across different cell types. Gene ontology (GO) enrichment analysis revealed that the *DMD* mutation activated the following four pathways in ECs: fibrillary collagen trimer, banded collagen fibril, complex of collagen trimers, and purine nucleotide metabolism. The study also found that the metabolic pathway activity of ECs was altered. Oxidative phosphorylation (OXPHOS), fatty acid degradation, glycolysis, and pyruvate metabolism were decreased while purine metabolism, pyrimidine metabolism, and one carbon pool by folate were increased. Moreover, the study investigated the impact of the *DMD* mutation on ECs from skeletal muscles and found a significant decrease in their overall number, but no change in their proliferation. Conclusions: Overall, this study provides new insights into the gene regulatory program in ECs in *DMD* and highlights the importance of further research in this area.

## 1. Introduction

The lack of the protein DYSTROPHIN caused by mutations in the *dystrophin* gene results in Duchenne muscular dystrophy (*DMD*), a genetic disorder that affects the cardiac and skeletal muscle system [1]. *Dystrophin* plays a crucial role in linking the sarcomere and the extracellular matrix and stabilizing the *dystrophin*-associated glycoprotein complex during muscle contraction and relaxation [2]. The progressive muscle cell atrophy caused by the absence of *dystrophin* leads to mobility loss and heart or lung failure in early childhood [3]. Cardiac abnormalities, such as dilated cardiomyopathy, arrhythmias, and congestive heart failure are common in patients with *DMD* [4]. Medical treatments such as corticosteroids and various drugs, including ACE inhibitors, beta-blockers, and antiarrhythmics, can help alleviate symptoms and delay the progression of heart failure [3]. Although *dystrophin* has been detected in vascular endothelial cells (ECs) and endothelial abnormalities in the response to shear stress have been observed in a mouse model of *DMD* [5], there is limited knowledge about the impact of *dystrophin* deficiency on the gene program of ECs. To shed light on this issue, we conducted a transcriptomic analysis of skeletal muscle-derived endothelial cells in *DMD*-mutant (*DMD^mut^*) and wild-type control mice using single-nuclear RNA sequencing (snRNA-seq) datasets.

Our snRNA-seq analysis revealed that skeletal muscle-derived ECs expressed the *dystrophin* gene. Through a systematic comparison of muscle-derived ECs from *DMD* mutant mice and control mice, we identified key target genes of *DMD* in ECs and discovered that the *DMD* mutation significantly altered pathways involved in the regulation of fibrillary collagen trimer, banded collagen fibril, complex of collagen trimers, and purine nucleotide metabolism. The snRNA-seq analysis also showed differences in the metabolic pathways of muscle-derived ECs from *DMD^mut^* mice compared to control mice. We also studied the impact of *DMD* mutation on ECs from skeletal muscles and showed a reduction in EC density but no effect on their proliferation. Overall, this study provides insight into the *DMD*-mediated gene regulation network in ECs.

## 2. Materials and Methods

### 2.1. SnRNA-Seq Datasets

The snRNA-seq data (barcodes, features, and matrix of gene expression) were downloaded from NCBI Gene Expression Omnibus (GEO) public database (GSE156498) [6]. In this snRNA-seq dataset, nuclei were isolated from TA muscle of ΔEx51 *DMD^mut^* mice and WT mice at 4 wk of age.

### 2.2. SnRNA-Seq Data Analysis

Seurat R package (V4.2) was used for downstream analytic procedures. Cells with extreme feature counts (<200 or >2500) and >20% reads with mitochondrial alignment were removed. Subsequently, we performed data normalization, high-variance feature identification, data scaling, and principal component analysis (PCA) using Seurat’s classic workflow. Then, the Harmony algorithm was used to correct the batch effects among samples. Next, dimensional reduction was performed using uniform manifold approximation and projection (UMAP) with the parameter “reduction” set as “harmony”. Seurat’s “FindNeighbors” and “FindClusters” functions were applied to the cell clustering analysis. Endothelial cells (ECs) were annotated according to the EC lineage marker gene PECAM1 (CD31) and the EC cluster was used for downstream analysis. EC cluster was further reclustered to generate 9 cluster subset (0–8) under wide-type and *DMD* conditions. To identify differentially expressed genes (DEGs) between *DMD^mut^* ECs and control ECs, the “FindMarkers” function under the default Wilcoxon rank sum test was applied to identify DEGs with avg_log2FC > 1 and *p*_val_adj < 0.05 as significant differential abundance. Volcano plots were generated using the R package “EnhancedVolcano” (V1.14). In addition, we also annotated skeletal muscle cells (SKM), and the SKM cluster was also used for downstream analysis to compare the major differentially expressed genes (DEGs) in SKM between *DMD^mut^* and WT mice.

### 2.3. Gene Ontology (GO) and GSEA Enrichment Analysis

We applied the gseGO function from R/Bioconductor “clusterProfiler package” (V4.4.4) and org.Mmu.eg.db (V3.15) to perform gene ontology (GO) pathway enrichment analysis and gene set enrichment analysis (GSEA) of DEGs with default parameters. The terms with *p* values < 0.05 identified from GO pathway enrichment analysis were considered significant. GO enrichment analyses were visualized as bubble plots, and the network of most enriched terms was visualized by cnetplot function.

### 2.4. Single-Cell Metabolic Analysis

To discern the metabolic difference between *DMD^mut^* and control ECs in snRNA-seq datasets, we applied the “scMetabolism” package to quantify the metabolic activities of individual ECs in *DMD^mut^* versus wild-type control mice. Specifically, the method was set to “VISION”, and analyzed using the Kyoto encyclopedia of genes and genomes (KEGG) metabolic gene sets.

### 2.5. Histology

To determine the vessel density and proliferation of ECs in the skeletal muscles of both *DMD^mut^* and control mice, we harvested skeletal muscles from 12-month-old male D2. B10-Dmd^Mdx^/J mice (*DMD^mut^*) and control DBA/2J mice (control) (The Jackson Laboratory, ME). Animals were handled according to approved protocols (2013-0537) and animal welfare regulations of the Institutional Animal Care and Use Committee of the Medical College of Georgia/Augusta University. We performed double immunostaining for both Ki-67 and CD31 in mouse skeletal muscles. Briefly, mouse muscles were paraffin-fixed, sectioned, and mounted on slides, and the paraffin-fixed sections were treated with xylene and a graded ethanol series to remove paraffin and rehydrate the tissue. After rehydration, heat-induced epitope retrieval was performed in 10 mM citrate buffer (pH 6.0). The tissue was then subjected to 5% goat serum blocking and streptavidin/biotin blocking (Vector Laboratories, Inc. Burlingame, CA). Heart sections were then stained overnight at 4 °C with biotinylated anti-mouse/rat Ki67 (1:100 dilution, eBioscience) and rabbit anti-CD31 (1:100 dilution, Cell Signaling Technology). The slides were incubated with anti-rabbit secondary antibody conjugated to Alexa 555 and streptavidin Alexa Fluor 488 conjugate (1:400 dilution, Life Technologies, Carlsbad, CA). Finally, the slides were mounted using VECTASHIELD HardSet mount media with DAPI. The Staining was analyzed by a Leica STELLARIS confocal microscope (Leica, Wetzlar, Germany). CD31-positive cells per high-power-field (40× HPF) were manually counted. Proliferative ECs were defined as CD31+ cells with Ki67+ nuclei in each field.

### 2.6. Statistical Analysis

Endothelial cell density and proliferation were expressed as mean ± SEM and analyzed using unpaired *t* tests using Prism 9.41 software (GraphPad Software, LLC).

## 3. Results

### 3.1. Single Cell Transcriptomics Reveals Nine EC Clusters

**Figure 1:**
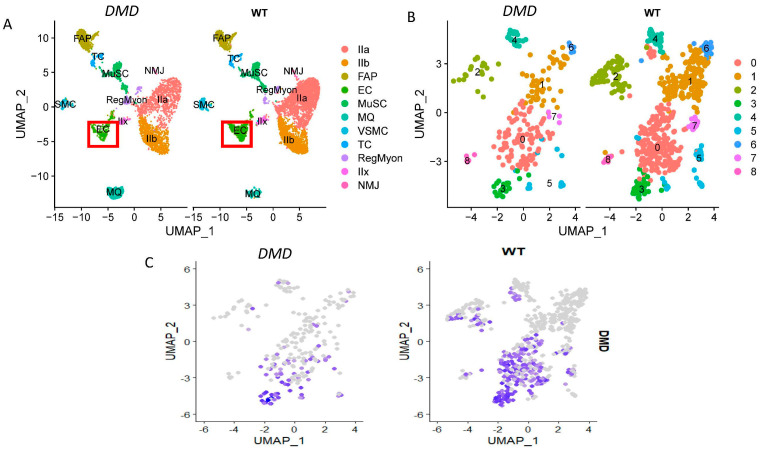
Single-nuclei RNA sequence analyses. (**A**) Split view of UMAP plot representation of muscles-derived nuclei from the *DMD^mut^* and wild-type (WT) mouse samples. Each major cell type was represented in a different color. A red square indicates EC subsets from muscle nuclei. IIa: type IIa myocytes, IIb: type IIb myocytes, IIx: type IIx myocytes, FAP: fibro/adipogenic progenitor cells, EC: endothelial cells, MuSC: muscle satellite cells, MQ: macrophage, VSMC: vascular smooth muscle cells, RegMyon: regenerative myocytes, TC: Tenocytes, and NMJ: neuromuscular junction myocytes. (**B**) Split view of UMAP plot representation of the ECs from *DMD^mut^* and WT mouse samples. (**C**) The colored expression levels of *DMD* in EC population from *DMD^mut^* and WT mouse samples were visualized in low-dimensional space with UMAP.

We performed snRNA-seq analyses on muscle tissues from *DMD^mut^* and control mice. Through unsupervised clustering, we determined the cellular composition of the muscle-derived cells, including type II myocytes (IIa, IIb, and IIx), fibro/adipogenic progenitor cells (FAP), endothelial cells (ECs), muscle satellite cells (MuSC), macrophage (MQ), vascular smooth muscle cells (VSMC), regenerative myocytes (RegMyon), Tenocytes (TC), and neuromuscular junction myocytes (NMJ) based on their respective molecular markers [6] (Figure 1A). To examine the presence of *DMD* in ECs, we subsetted EC clusters and found that the total number of ECs was lower in *DMD^mut^* muscles (235 ECs) compared to control muscles (627 ECs), suggesting that the *DMD* mutation may have a negative impact on the number of ECs in skeletal muscles. We identified 9 subpopulation of ECs (cluster 0–8) (Figure 1B). Our results revealed that *DMD* expression was significantly reduced in *DMD^mut^* ECs compared to control ECs (Figure 1C).

### 3.2. Differential Gene Expression and Functional Enrichment Analysis of ECs in DMD^mut^ versus Control Muscles

**Figure 2:**
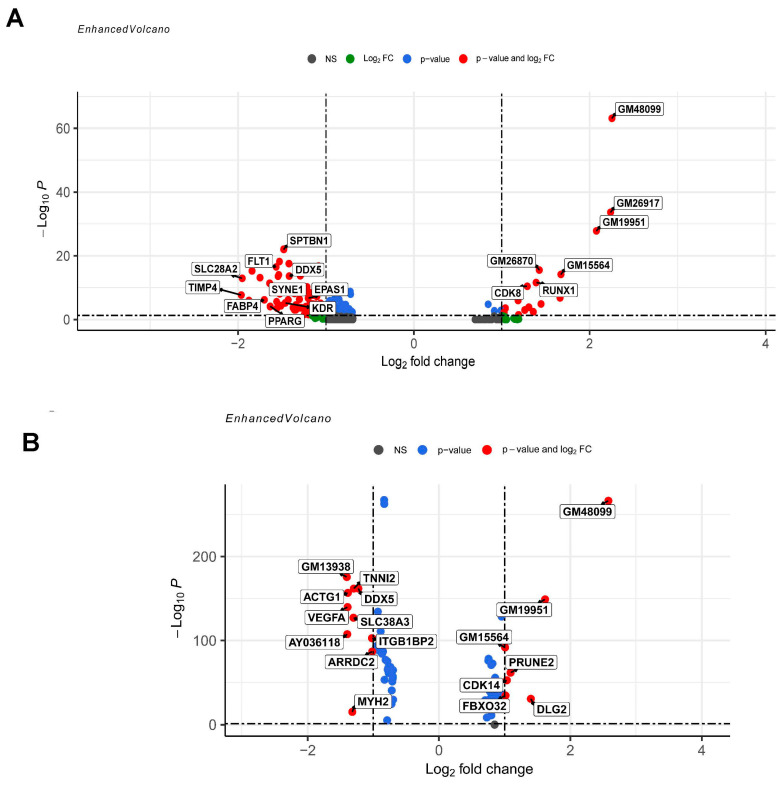
Differential gene expression in ECs and SKMs from *DMD^mut^* versus control muscles. (**A**) Differential gene expression in ECs from *DMD^mut^* versus control muscles. Volcano plots were used to show the adjusted *p* values and log2 fold change values of the genes in ECs from *DMD^mut^* and wild-type control muscles. Differentially expressed genes (DEGs), represented by red dots, were defined as genes with an adjusted *p* value of ≤0.05 and a log2 fold change value of greater than 0.26 in magnitude. Square symbols were used to indicate upregulated genes (such as GM48099, GM26917, GM19951, GM26870, GM15564, RUNX1, and CDK8) and a downregulated gene (such as SPTBN1). (**B**) Differential gene expression in SKMs from *DMD^mut^* versus control muscles. Volcano plots were used to show the adjusted *p* values and log2 fold change values of the genes in SKMs from *DMD^mut^* and wild-type muscles. DEGs, represented by red dots, were defined as genes with an adjusted *p* value of ≤0.05 and a log2 fold change value of greater than 0.26 in magnitude. Square symbols were used to indicate upregulated genes (such as GM48099, GM19951, GM15564, PRUNE2, CDK14, et al.) and downregulated genes (such as TNNI2, DDX5, VEGFA, MYH2, ITGB1BP2, SLC38A3, et al.).

**Figure 3:**
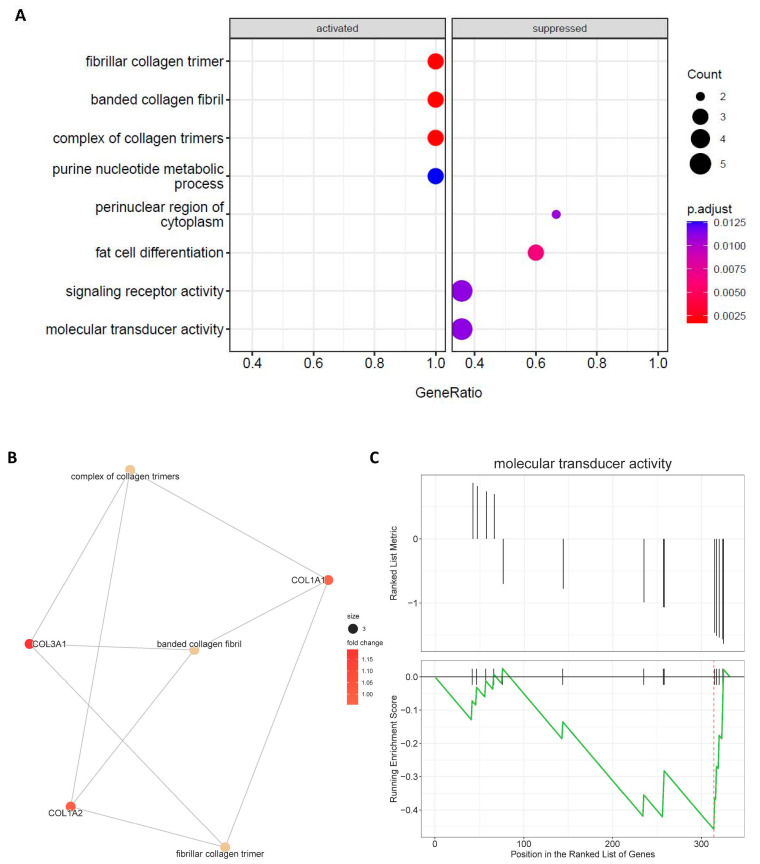
Functional enrichment analysis of ECs and EC-specific expression change of *DMD^mut^* and WT muscles. (**A**) Dot plot representing the top four gene ontology (GO) with the largest gene ratios in order of gene ratio. The color gradient of dots represents the adjusted *p* values, while the size represents the number of genes in the significant DEG list associated with the GO term. (**B**) The cnetplot depicts the linkages of genes and GO terms as a network that is helpful to see which genes are involved in enriched pathways. (**C**) GSEA plot of the running enrichment score (green line) for a gene set as the analysis walks down the ranked gene list, including the location of the maximum enrichment score (the red line). The black lines in the running enrichment score show where the members of the gene set appear in the ranked list of genes, indicating the leading edge subset.

We performed differential gene expression analysis to compare ECs in *DMD^mut^* and control muscle and found that *DMD^mut^* ECs had significantly elevated levels of various long noncoding RNAs (lncRNAs), including GM48099, GM26917, GM19951, GM15564, and GM26870. Additionally, CDK8 and RUNX1 were also significantly upregulated in *DMD^mut^* ECs (Figure 2A). CDK8 is a cyclin-dependent kinase that regulates the transcriptional activity of RNA polymerase II [7]. RUNX1, on the other hand, is a critical transcription factor that plays a role in hematopoiesis [8]. It is essential for the hematopoietic commitment of hematopoietic ECs and it converts ECs into hematopoietic progenitor cells [9]. Next, we compared the major DEGs in skeletal muscle cells (SKMs) between *DMD^mut^* and control muscles. The SKM dataset contained 1837 cells from *DMDmut* and 4078 cells from control muscles. By comparing the DEGs between Figure 2A,B, we found that certain lncRNAs, such as GM48099, GM19951, and GM15564, were consistently upregulated in ECs and SKMs from *DMD^mut^* ECs and SKMs, indicating that these dysregulated lncRNAs are conserved across different cell types. Furthermore, we also found that DDX5 was downregulated in both *DMD^mut^* ECs and SKMs from *DMD^mut^*, suggesting that the repression of DDX5-mediated gene regulation is independent of cell types. We conducted a gene ontology (GO) enrichment analysis using the clusterProfiler package and found that the four most significantly activated pathways in *DMD^mut^* ECs are related to fibrillary collagen trimer, banded collagen fibril, complex of collagen trimers, and purine nucleotide metabolism (Figure 3A). These findings highlight the important role of *DMD* in regulating lncRNAs and EC fate.

The gene expression analysis revealed that a number of genes were significantly downregulated in *DMD^mut^* ECs, including SPTBN1, TIMP4, SLC28A2, FABP4, SYNE1, PPARG1, FLT1, KDR, DDX5, EPAS1, etc. (Figure 2A). FLT1 and KDR, two VEGF receptors, are involved in the signaling of VEGF in vascular ECs [10]. Endothelial PAS domain protein 1 (EPAS1) is a transcription factor specific to ECs and has a role in regulating EC fate [11]. Tissue inhibitor of metallopeptidase 4 (TIMP4), which has been shown to inhibit capillary EC migration, is not a critical regulator of EC function, such as EC proliferation and angiogenesis in vivo [12]. Slc28a2 is a purine nucleobase transmembrane transporter important for maintaining intracellular nucleotide levels and preventing apoptosis [13]. Fatty acid-binding protein 4 (FABP4), an intracellular lipid chaperone induced by VEGF in some ECs, influences angiogenesis through its effect on gene expression [14]. DDX5, a well-known multifunctional DEAD-box RNA helicase and a transcription cofactor [15], is involved in Notch signal. Lin S. et al. [16] reported that DDX5 enhanced Notch-mediated transcription, and DDX5 depletion inhibited Notch signature gene expression in leukemic cells. Peroxisome proliferator-activated receptor γ 1(PPARG1), an isoform of PPARG generated through splicing, controls lipogenesis in mammary cells [17]. These downregulated genes play a role in the angiogenesis of ECs.

The GO term analysis revealed that the pathways, which are most significantly suppressed in *DMD^mut^* ECs, are mainly related to the perinuclear region of the cytoplasm, fat cell differentiation, signaling receptor activity, and molecular transduction (Figure 3A). To understand the complex relationship between the enriched pathways, we used the cnetplot function. This allows the visualization of the genes involved in the enriched pathways and genes belonging to annotation categories. Figure 3B shows the network of the most enriched upregulated genes (COL3A1, COL1A2, and COL1A1) involved in banded collagen fibrils, complex of collagen trimers, and fibrillary collagen trimers. To identify top pathway responsible for the observed phenotypes in *DMD^mut^* and control ECs, we performed the gene set enrichment analyses (GSEA). The top gene set enriched in the *DMD^mut^* ECs compared to control ECs was a classifier for inhibited molecular transducer activity (Figure 3C).

### 3.3. Differential Metabolic Pathways of ECs within DMD^mut^ and Control Muscles

**Figure 4:**
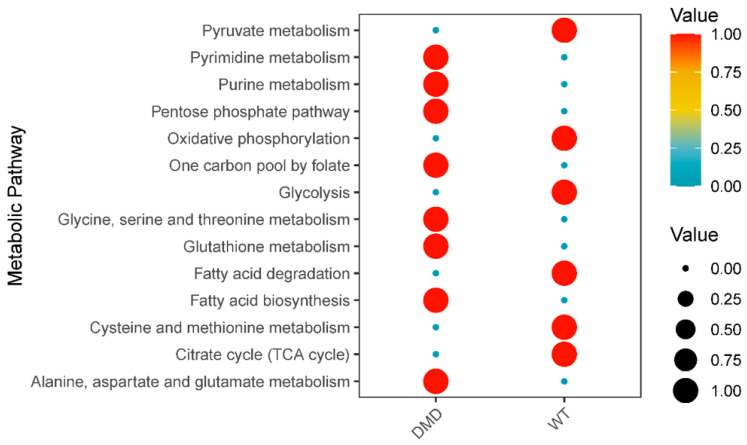
Dot plot illustration of the metabolic pathway analyses performed by “scMetabolism” R package for ECs from *DMD^mut^* and WT muscle.

We investigated the metabolic changes in ECs after *DMD* mutation using the “scMetabolism” package, which covers a wide range of metabolic pathways. The metabolic pathways were filtered, and the activity was compared between ECs from *DMD^mut^* and control muscles. The results indicated significant differences in multiple metabolic pathways:

(i) ECs are more glycolytic and less dependent on oxidative phosphorylation (OXPHOS), despite having punctate mitochondria [18]. Recent studies demonstrated that both OXPHOS and glycolysis are essential for the angiogenic response of vascular ECs [19]. “scMetabolism” analysis revealed that ECs from *DMD^mut^* showed lower activities of oxidative citric acid cycle (TCA cycle), OXPHOS, fatty acid degradation, glycolysis and pyruvate metabolism compared to ECs from control muscles (Figure 4);
(ii) Although the role of fatty acid synthesis in ECs remains incompletely described, recent study reported that fatty acid synthase knockdown in ECs impedes vascular sprouting by reducing proliferation. Loss of fatty acid synthase in ECs also impairs angiogenesis in vivo [20]. Our scRNA-seq analysis revealed that ECs from *DMD^mut^* showed high activity of fatty acid biosynthesis, which has been linked to vascular sprouting and angiogenesis (Figure 4);
(iii) Trophic effects of purine as well as pyrimidine nucleosides and nucleotides promote migration and proliferation of ECs via P1 and P2Y receptors during angiogenesis, vascular remodeling, and atherosclerosis during restenosis after angioplasty [21,22]. One-carbon folate pool is needed for the synthesis of purines and thymidylate in nucleic acids [23]. “scMetabolism” analysis revealed that ECs from *DMD^mut^* muscles showed increased activities of purine metabolism, pyrimidine metabolism, and one carbon pool by folate, which play a role in promoting migration and proliferation of ECs during angiogenesis and restenosis (Figure 4);
(iv) Recent studies demonstrated the role of the endothelial oxidized pentose phosphate pathway (oxPPP) in promoting cell coverage and a maturation of the vascular wall by controlling vascular matrix composition and vascular mural cell recruitment during early development. These findings establish a key role for oxPPP in the formation of the vascular system [24,25]. “scMetabolism” analysis shows that ECs from *DMD^mut^* muscle showed high pentose phosphate pathway activity, which plays a role in the formation of the vascular system (Figure 4);
(v) Metabolism of serine, glycine, and threonine provides energy for the TCA cycle via pyruvate and acetyl CoA [26]. Glutathione (GSH) plays an important role in the cellular defense against peroxidative stress. In amino acid metabolic pathways, homocysteine is an intermediate amino acid that is metabolized from methionine to cysteine and has been shown to be associated with oxidative stress and endothelial damage [27]. Glutathione (GSH), superoxide dismutase (SOD), and glutathione peroxidase (GPX) are major antioxidant molecules in ECs [28]. A recent report showed that brain ECs directly absorb and metabolize glutamate and utilize the resulting α-ketoglutarate in the tricarboxylic acid cycle, ultimately producing ATP in the absence of glucose [29]. Our “scMetabolism” analysis shows that ECs from *DMD^mut^* showed high activities of glycine, serine, and threonine metabolism, glutathione metabolism, and alanine, aspartate, and glutamate metabolism, but low activity of cysteine and methionine metabolism compared to ECs from control muscle (Figure 4).

### 3.4. Impact of DMD Mutation on EC Density and Proliferation in Skeletal Muscles

**Figure 5:**
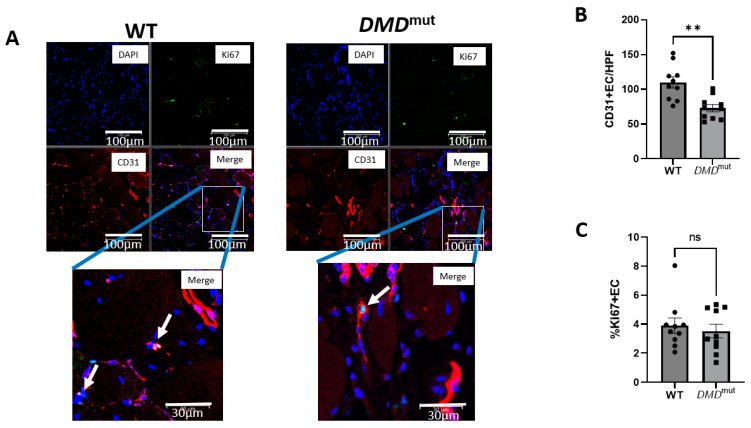
The effect of *DMD* mutation on the density and proliferation of endothelial cells in *DMD^mut^* muscles compared to control muscles. (**A**) Immunofluorescent staining of CD31 and Ki67 was performed to detect EC density and cell proliferation in TA muscles from *DMD^mut^* and wide-type control mice. The regions of interest (white squares) were cropped from images (Upper). The CD31/Ki67-double positive cells were indicated with arrows. (**B**) The comparison of CD31-positive cells per high power field (HPF) between TA muscles from *DMD^mut^* and control mice (**, *p* <  0.01, n  = 10). (**C**) The comparison of the percentage of Ki67-positive EC cells between TA muscles from *DMD^mut^* and wide-type control mice (ns, n  =  10).

Our analysis of SnRNA-seq data indicated a reduction in the overall number of ECs in muscles with the *DMD* mutation compared to control muscles. To investigate this further, we evaluated EC density in the tibial anterior (TA) muscles of both *DMD^mut^* and control mice using CD31 immunofluorescence staining. The results showed a significant decrease in EC density in the muscles of *DMD^mut^* mice compared to the control muscles (Figure 5A,B). To determine the effect of the *DMD* mutation on EC proliferation, we conducted double immunofluorescence staining for Ki67 and CD31. Although there was no significant difference in the number of proliferative ECs (indicated by Ki67+) in *DMD^mut^* muscles compared to control muscles (Figure 5C), the *DMD* mutation still led to a decrease in the overall number of ECs.

## 4. Discussion

In this study, we analyzed snRNA-seq data from the skeletal muscles of *DMD^mut^* mice and control mice. The results showed that the most activated pathways in endothelial cells (ECs) from *DMD^mut^* were related to fibrillar collagen, while the most suppressed pathways were associated with signaling receptor activity and molecular transduction. Additionally, we observed a significant upregulation of multiple lncRNAs, RUNX1, and CDK3 in ECs from *DMD^mut^* as well as a downregulation of crucial genes for EC function. The *DMD* mutation also impacted major energy metabolism pathways, causing a downregulation of OXPHOS and glycolysis, and an upregulation of nucleotide acid metabolism. Further analysis showed that the *DMD* mutation reduces the overall number of ECs in skeletal muscles but does not alter EC proliferation.

Spectrin is a protein that provides structure to cell membranes and is recognized for its vital role in preserving the shape of cells and shielding them from mechanical stress. Spectrin Beta, Non-Erythrocytic 1 (SPTBN1), which plays a role in regulating cell shape, DNA damage repair, angiogenesis, and stemness, is critical for the regulation of DNA damage repair, angiogenesis, and stemness maintenance [30]. Recent study demonstrated that spectrin responds dynamically to forces during cell–cell fusion, forming a transient, spectrin-rich network at the fusion site. This network acts as a barrier to restrict adhesion molecules and a filter for invasive protrusions, helping to create the conditions for successful membrane fusion [31]. Interestingly, McCount JL et al. [32] found that sarcospan overexpression in *DMD^mut^* skeletal muscle can rescues dystrophin deficiency partially through mechanotransduction signaling cascades mediated through a rewiring of cell-extracellular matrix (ECM) bidirectional communication, along with spectrin beta by multi-omics analysis.

In a previous study, it was found that *dystrophin* is expressed in vascular ECs as seen through confocal immunostaining in isolated perfused arteries and plays a crucial role in regulating shear stress and vascular dilation in cardiac muscles of mice [5]. Mdx mice had an abnormal mechanotransduction of shear stress in both large and resistance arteries [5], as well as lower nitric oxide (NO)-dependent flow (shear stress)-mediated endothelium-dependent dilation and decreased vascular density in cardiac muscles compared with control mice, implying the crucial role of endothelial *dystrophin* in vascular dilation [33]. However, another study found that, while the vasodilation response was impaired in Mdx mice, the response to acetylcholine or sodium nitroprusside remained unchanged [34].

The relationship between *DMD* and angiogenesis is controversial, with different studies yielding conflicting results. For instance, Kunert-Keil C et al. [35] found no significant difference in muscle angiogenesis in Mdx mice, with no alterations in VEGF mRNA and protein levels. However, Loufrani L et al. [33] reported a decrease in vascular density in the cardiac muscles of Mdx mice compared to controls. Some studies have even shown increased angiogenesis in the brain of Mdx mice, potentially due to factors such as HIF-1α activation, VEGF, and VEGFR-2 upregulation, and changes in ZO-1 and claudin-1 [36,37,38]. In a recent study by Podkalicka P et al. [39], the impact of *DMD* on angiogenesis was found to be age-dependent. The researchers used a hindlimb ischemia (HLI) model to evaluate angiogenesis/arteriogenesis processes in wild-type and Mdx mice. They discovered that young Mdx mice had decreased VEGFA levels in various muscle types, but the abundance of CD31/α-SMA double-positive blood vessels and basal blood flow were unchanged. However, blood flow was reduced, and there was an increase in the number of CD31/α-SMA double-positive blood vessels in old Mdx mice, suggesting that aging affects blood vessel function in Mdx mice and that older Mdx mice can serve as an appropriate model for testing the effectiveness of vascular-based therapies aimed at restoring functional angiogenesis to alleviate *DMD* severity [39].

To resolve these conflicting findings, it is essential to examine the expression of *DMD* in the ECs of muscle tissues and to uncover the gene regulatory network that is controlled by *DMD* in these cells. SnRNA-seq allows for a comprehensive analysis of the transcriptome in individual cells, facilitating the detection of differentially expressed genes, major regulatory pathways, and co-expression networks among cell subpopulations. By using snRNA-seq data from both *DMD* mutant and wild-type mice, we were able to confirm the presence of *DMD* in ECs and identify nine EC subpopulation clusters. Our analysis revealed that several crucial genes involved in EC function, including TIMP4, SLC28A2, FABP4, SYNE1, PPARG1, FLT1, KDR, DDX5, SPTBN1, and EPAS1, were significantly downregulated in *DMD* mutant ECs compared to control cells. On the other hand, the expression of multiple long noncoding RNAs, including GM48099, GM26917, GM19951, GM15564, and GM26870, was upregulated in ECs from *DMD^mut^*. Our snRNA-seq data analysis also showed a lower number of ECs in *DMD^mut^* muscles compared to control muscles, indicating a possible negative impact of the *DMD* mutation on the quantity of ECs in skeletal muscles. CD31 immunofluorescence staining further confirmed this, demonstrating a significant decrease in EC density in *DMD^mut^* mice as compared to control mice. The absence of a difference in the proliferation of ECs, as indicated by Ki67+ CD31+ cells, is a noteworthy finding. It suggests that the *DMD* mutation does not directly impact the proliferative ability of ECs, which has implications for understanding the underlying mechanisms of the reduced number of ECs in *DMD^mut^* muscles. This analysis provides important insights into the role of *DMD* in ECs and highlights the potential for using snRNA-seq to study gene regulation in these cells.

The results of our snRNA-seq gene ontology pathway analysis showed that the *DMD* mutation resulted in an increased formation of collagen fibrils in ECs, including fibrillary collagen trimers, banded collagen fibrils, and complexes of collagen trimers. Dystrophy in *DMD* is characterized by muscle cell loss and fibro-fatty replacement [40,41,42]. Normally, the tissue collagen balance is maintained, but in some pathological conditions, this balance shifts toward fibrosis, which involves the excessive production and accumulation of collagen. To reduce the formation of fibrotic tissue in *DMD^mut^* muscles, preventing the assembly and cross-linking of collagen chains may be an effective strategy [43].

LncRNAs play a role in regulating various biological processes through a variety of mechanisms. They are transcribed by RNA polymerase II in a way that is dependent on the cell environment, and they possess features similar to mRNAs, such as 5′ caps, polyA tails, and splice sites [44]. These polyA-tailed lncRNAs can be detected using Chromium Single Cell 3′ 10× Genomics single-cell RNA sequencing (scRNA-seq), which captures RNAs based on their 3′-biased polyA tails [45]. A previous study using gene expression microarray identified 14 lncRNAs of chromosome X that were specifically transcribed by the *DMD* locus and may be involved in regulating *dystrophin* isoform transcription [46]. However, none of the lncRNAs that were significantly upregulated in ECs from *DMD^mut^* were from chromosome X (GM48099 from chromosome 14, GM26917 from chromosome 17, GM19951 from chromosome 12, GM15564 from chromosome 16, and GM26870 from chromosome 9). LncRNA GM26917, which has not been widely studied, has been shown to be induced by lipopolysaccharide (LPS) and to regulate global inflammatory gene expression and macrophage differentiation. Silencing GM26917 has been shown to protect against LPS-induced liver injury by regulating the Toll-like receptor 4 (TLR4)/NF-κB signaling pathway in macrophages [47]. The function of GM26870 is not yet well understood, but a recent study suggests that it may play a role in nicotinamide phosphoribosyltransferase (Nampt)-mediated pathways by forming extensive ribonucleoprotein (RNP) complexes with chromatin regulators. The role of these lncRNAs in the pathophysiology of the *dystrophic* ECs remains to be determined.

## 5. Conclusions

In conclusion, our snRNA-seq study showed that the *DMD* gene is expressed in muscle-derived ECs. Our comparison of muscle-derived ECs from *DMD^mut^* mice and control mice revealed key target genes of *DMD*. We showed that the *DMD* mutation affected fibrillary collagen trimer, banded collagen fibril, complex of collagen trimers, and purine nucleotide metabolism pathways. The snRNA-seq also indicated differences in the metabolic pathways of muscle-derived ECs between *DMD^mut^* mice and control mice. The study also showed a reduction in EC density in *DMD^mut^* muscle, but no effect on EC proliferation. This provides valuable insight into the *DMD*-regulated gene network in ECs.

## Data Availability

Not applicable.
